# Screening natural raw materials and product development for improving insomnia based on network pharmacology and data mining

**DOI:** 10.1097/MD.0000000000047072

**Published:** 2026-01-23

**Authors:** Lizhi Yue, Qian Jiao, Junxiang Li, Yi Lu, Meiting Yi, Congfen He, Yan Jia

**Affiliations:** a Key Laboratory of Cosmetic of China National Light Industry, Beijing Technology and Business University, Beijing, China; b School of Chemistry and Chemical Engineering, Qilu Normal University, Shandong, China; c AGECODE R&D Center, Yangtze Delta Region Institute of Tsinghua University, Zhejiang, China; d Research & Development Department, Harvest Biotech (Zhejiang) Co., Ltd, Zhejiang, China.

**Keywords:** Chinese herbal medicine, data mining, insomnia, network pharmacology, raw materials

## Abstract

Insomnia, as one of the most common sleep problems, seriously affects the normal life and work of individuals. Aromatherapy is regarded as a promising alternative medicine for improving sleep quality. Based on network pharmacology and data mining, this study screened natural raw materials for improving insomnia. Then, we developed an aromatherapy product informed by the screening results and investigated its mechanism for improving insomnia through network pharmacology. Five core insomnia targets were identified through literature. 1600 candidate compounds and 1757 candidate herbs related to the target were matched using HERB and TCMSP databases. By comparing with the Catalogue of Used Cosmetic Materials (2021 edition), 597 kinds of usable candidate materials were selected, including 85 raw materials related to target MTNR1A, 86 raw materials related to target MTNR1B, 120 raw materials related to target HTR1A, 7 raw materials related to target GABRB2, and 582 raw materials related to target GABRA1. Then based on the screening results, we selected sandalwood, lime, angelica sinensis, yilan, sage and lavender to design Pinghe Sleep Aromatherapy Product to improve insomnia. Network pharmacological analysis revealed that the main ingredients of the Pinghe Sleep Aromatherapy Product are beta-sitosterol, stigmasterol, isorhamnetin, luteolin, tanshinone IIA, D-limonene, and linalool. It exerts improvement effects by influencing targets such as IL6, TNF, AKT1, CASP3, TP53, and VEGFA, regulating signaling pathways such as AGE-RAGE, neuroactive ligand-receptor interactions, the HIF-1 signaling pathway, and the calcium signaling pathway. This study provides an idea of raw material screening and product development, which can save product development cost and shorten product development cycle by using network pharmacology and data mining.

## 1. Introduction

Insomnia refers to a subjective experience of inadequate sleep duration or quality despite suitable sleep opportunities and sleep environments and affects social functioning during the day. The main symptoms include difficulty falling asleep (sleep latency >30 minutes), sleep maintenance disorder (≥2 awakenings throughout the night), early waking, decreased sleep quality and total sleep time (usually <6.5 hours), accompanied by daytime dysfunction. Daytime dysfunction caused by insomnia mainly includes fatigue, depression or irritation, physical discomfort, cognitive impairment, etc.^[1]^ Long-term insomnia affects an individual’s normal life and work, increases the risk of various health problems,^[2]^ and may impair brain neuroplasticity and stress immune pathways, leading to mental disorders.^[3]^ It may even cause serious accidents and endanger people’s life safety.^[4]^ Insomnia is one of the most common sleep problems and is clinically common, occurring in up to 50% of primary care patients. Insomnia can occur independently or in conjunction with other diseases or mental health disorders.^[5]^ People with anxiety or depression, women, the elderly, and people with chronic conditions such as asthma and chronic obstructive pulmonary disease are at greater risk for insomnia.^[6]^ Restoring good sleep continuity and longer sleep duration can improve sleep-related functions, including mood, memory, cognition, weight, blood pressure, glucose regulation, amyloid beta clearance, and immune function.^[5]^ Many sleep-regulating substances are involved in circadian rhythms and sleep regulation. Studies have shown that endogenous molecules related to insomnia mainly include γ-aminobutyric acid (GABA), adenosine, serotonin, melatonin, and prostaglandin D2.^[7]^ At present, the main intervention methods for insomnia include drug therapy, psychological therapy, physical therapy, and Chinese ethnic medicine therapy. The short-term efficacy of drugs for insomnia has been confirmed by clinical trials, but long-term application still needs to bear the potential risks of drug adverse reactions, addiction and so on. Aromatherapy, massage, and other methods also have their unique application advantages in the treatment and improvement of insomnia.^[1]^

Aromatherapy is regarded as a promising alternative medicine for improving sleep quality. Aromatic components have the function of nourishing the heart and calming the mind, regulating qi and resolving depression, and have a long history of use in China.^[8,9]^ As a daily consumer product, aromatic products have the advantages of frequent use and long-term use without side effects. Network pharmacology is a scientific idea and research strategy based on high-throughput omics data analysis, computer virtual computing, biological information network construction and network topology analysis strategies and technologies.^[10]^ This study was based on network pharmacology and data mining to screen Chinese characteristic natural raw materials for improving insomnia. Then based on the screening results, we designed Pinghe Sleep Aromatherapy Product (PSAP), and used network pharmacology to explore the key components and core targets of PSAP in improving insomnia, as well as the action pathways of components and targets. Our findings provide novel insight and data reference for the development of aromatic products for improving insomnia.

## 2. Materials and methods

### 2.1. Network pharmacology screens natural raw materials for improving insomnia

#### 2.1.1. Collect potential targets for insomnia

The Traditional Chinese Medicine Systems Pharmacology Database and Analysis Platform (TCMSP) (https://www.tcmsp-e.com/tcmsp.php) was searched with “insomnia” and “chronic insomnia” as keywords, and the targets of insomnia were collected, and then the main targets of insomnia were determined by supplementing the results of literature search.

#### 2.1.2. Screening candidate compounds and Chinese herbs

Compounds and Chinese herbs related to the target were collected by searching the TCMSP platform and the High-throughput Experimental and Reference guided database of traditional Chinese medicine (HERB) (http://herb.ac.cn), and repetitive components were deleted to obtain the target-related compounds set and Chinese herbs set.

#### 2.1.3. Selection of available raw materials for candidate cosmetics

The above Chinese herbal medicine raw materials were compared with the Catalogue of Used Cosmetic Raw Materials (2021 edition) one by one to screen the available cosmetic raw materials.

#### 2.1.4. Network construction

The network diagram of target-compound – raw material is constructed by using Cytoscape 3.9.1 (California), and the topological parameters of nodes in the network were calculated. According to the degree value of each node in the network, the key node was determined, and the core compound and the available raw material were determined.

### 2.2. Network pharmacological analysis of the improvement of insomnia by PSAP

#### 2.2.1. Insomnia related target screening

Three databases were used to screen the potential targets of insomnia. They were the TCMSP (https://old.tcmsp-e.com/tcmsp.php), Genecards database (https://www.genecards.org/) and Disgenet database (http://www.disgenet.org). Merged the collected targets and deleted duplicate data.

#### 2.2.2. Prediction of target of PSAP

PSAP is composed of sandalwood, lime, angelica sinensis, yilan, sage and lavender. Potential targets of these raw material were screened using TCMSP (https://old.tcmsp-e.com/tcmsp.php) and supplemented by literature search using PubMed. Merged the collected targets and deleted duplicate data.

#### 2.2.3. Prediction of therapeutic targets of PSAP in the improving of insomnia

The online Venn analysis tool (https://bioinfogp.cnb.csic.es/tools/venny/) was used to calculate and draw custom Venn diagrams to obtain the intersection targets of PSAP in the improving of insomnia.

#### 2.2.4. Construction of the PPI network and screening of core targets

Import the intersection target into the Search Tool for Recurring Instances of Neighbouring Genes platform (https://cn.string-db.org) for analysis. The species is limited to Homo sapien. Create a protein–protein interaction (PPI) network, download the PPI network, and import into Cytoscape 3.9.1 to perform core target analysis based on Centiscape 2.2 (California) plug-in. The core target whose closeness, betweenness and degree are greater than the central value is the core target.

#### 2.2.5. GO and KEGG pathway analysis

Import the intersection targets into the Metascape database (https://metascape.org/gp/index.html#/main/step1) for gene ontology (GO) and Kyoto Encyclopedia of Genes and Genomes (KEGG) pathways analysis. The organism selected was Homo sapiens. The significance level was set to *P* < .05, and the top 20 analysis results were plotted respectively.

## 3. Results

### 3.1. Network pharmacology screens natural raw materials for improving insomnia

#### 3.1.1. Target acquisition

Through literature research and TCMSP database search, 5 main targets for insomnia were identified as shown in Table 1.

**Table 1:** Information of potential targets.

Gene symbol	Uniprot ID	Protein name
MTNR1A	P48039	Melatonin receptor type 1A
MTNR1B	P49286	Melatonin receptor type 1B
HTR1A	P08908	5-Hydroxytryptamine receptor 1A
GABRA1	P14867	Gamma-aminobutyric acid receptor subunit alpha-1
GABRB2	P47870	Gamma-aminobutyric acid receptor subunit beta-2

#### 3.1.2. Acquisition of candidate compounds and Chinese herbs

HERB and TCMSP were used to retrieve components associated with 5 targets. Considering that aromatic products are used for external use only and are not used in the same way as drugs, they are not suitable for the pharmacokinetic parameters and Lipinski rule referenced in drug screening.^[11]^ In this study, no screening parameters were set when screening compounds, and all the compounds related to the target were collected, and a total of 1600 compounds were obtained. HERB and TCMSP were used to search the Chinese herbs related to the compounds. A total of 12,950 Chinese herbs were collected, and 1757 kinds of Chinese herbs were obtained after weight removal.

#### 3.1.3. Availability of raw materials for candidate cosmetics

The above 1757 candidate Chinese herbs were compared with the raw materials in the Catalogue of Used Cosmetics Raw Materials (2021 edition). In the screening, the common name and alias of the raw material should be considered comprehensively to avoid omission. A total of 597 candidate raw materials for improving insomnia were obtained.

#### 3.1.4. Target-compound – raw material network construction

##### 3.1.4.1. Network construction of target MTNR1A

**Figure 1.**
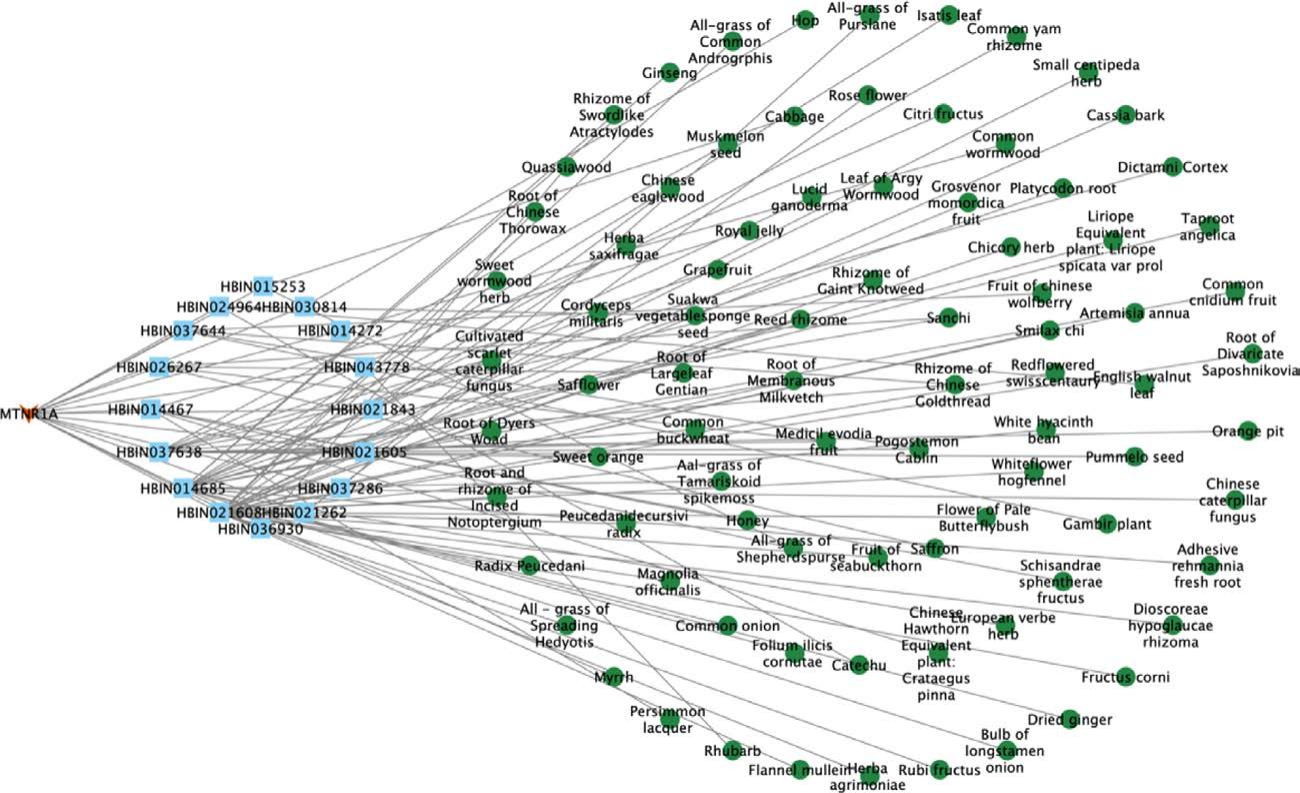
Target-compound- candidate raw materials network of MTNR1A. MTNR1A = melatonin receptor 1A.

**Figure 2.**
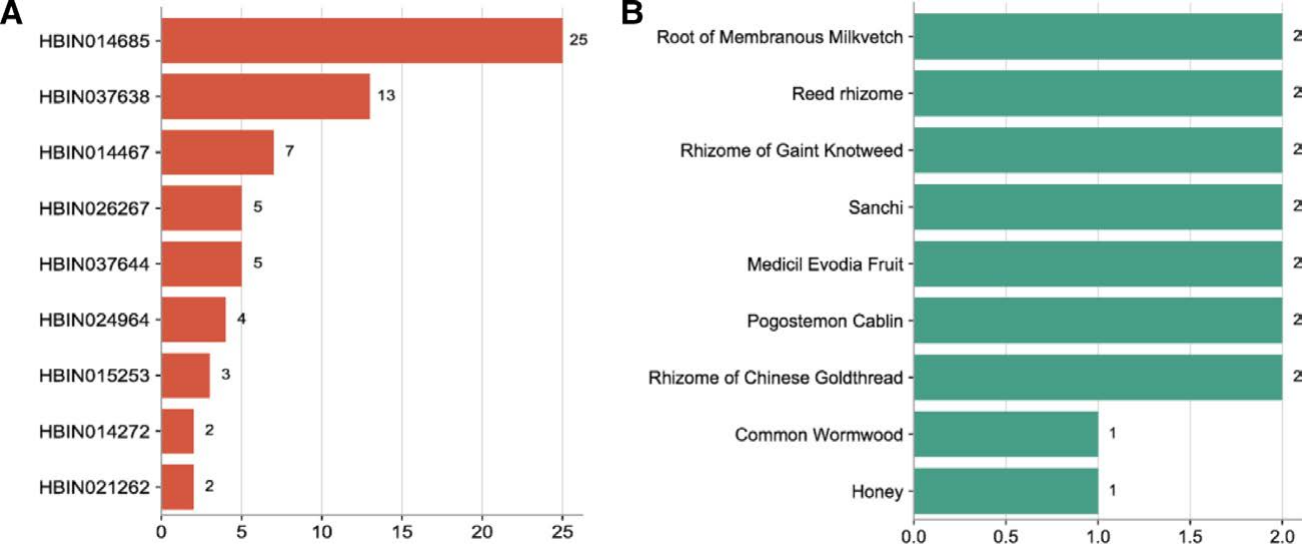
Compounds and candidate raw materials related to target MTNR1A. (A) Top 10 compounds with degree; (B) top 10 candidate raw materials with degree. MTNR1A = melatonin receptor 1A.

Through screening, 16 compounds related to the target MTNR1A, and 85 kinds of related raw materials were obtained. The network diagram constructed by the target, candidate compounds and candidate raw materials was shown in Figure 1. According to the size of the degree value, take the top 10 compounds and candidate raw materials to make a bar chart, as shown in Figure 2A and B. The top 5% candidate raw materials with the degree value are taken as the core raw materials. The core raw materials related to the target MTNR1A in terms of degree value were root of membranous milkvetch, reed rhizome, rhizome of gaint knotweed, sanchi, medicil evodia fruit. The annotations of the compounds are shown in Table 2.

**Table 2:** Notes on compounds associated with the target MTNR1A.

Number	Compound ID	Compound name
1	HBIN014272	Absinthin
2	HBIN014467	Acetylcholine
3	HBIN014685	Adeninenucleoside
4	HBIN015253	Allyl isothiocyanate
5	HBIN021262	Colchine
6	HBIN021605	Coumari
7	HBIN021608	Coumarin
8	HBIN021843	Cucurbitacin
9	HBIN024964	Elaterin
10	HBIN026267	Evodin
11	HBIN030814	Isohumulone A
12	HBIN036930	Nigakilactone D
13	HBIN037286	(−)-Noradrenaline
14	HBIN037638	Obaculactone
15	HBIN037644	Obakulactone
16	HBIN043778	Serotonine

##### 3.1.4.2. Network construction of target MTNR1B

**Figure 3.**
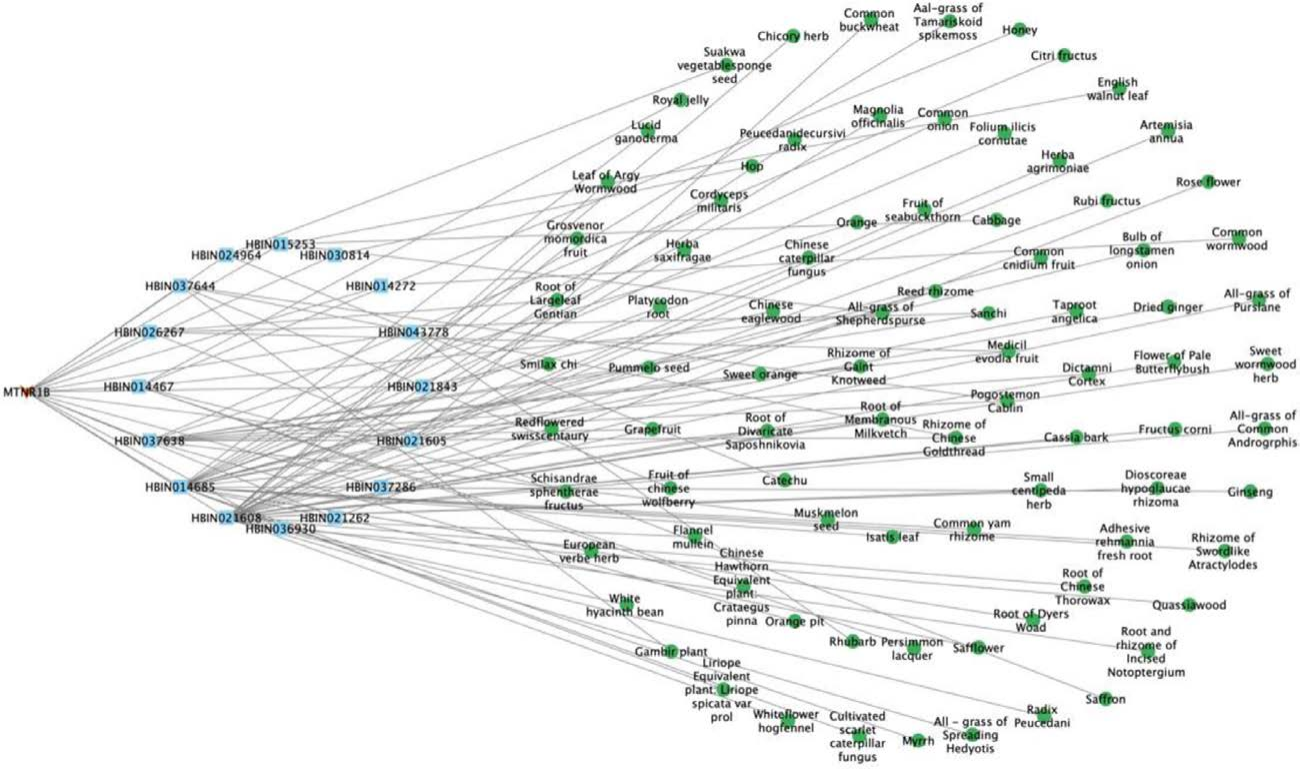
Target-compound- candidate raw materials network of MTNR1B. MTNR1B = melatonin receptor 1B.

**Figure 4.**
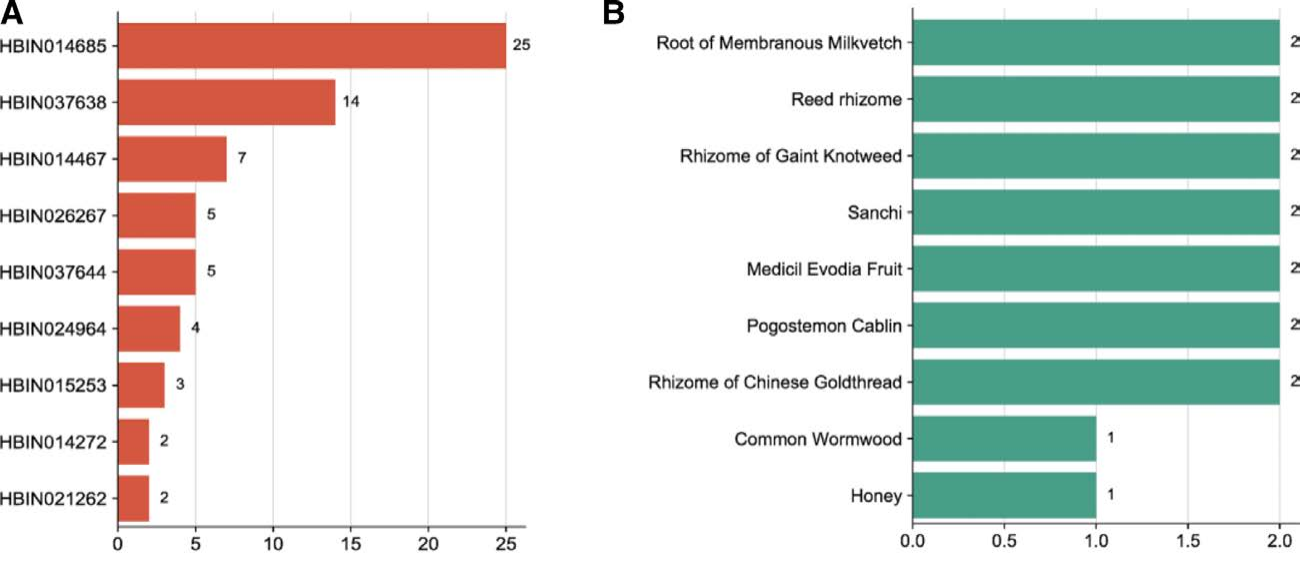
Compounds and candidate raw materials related to target MTNR1B. (A) Top 10 compounds with degree; (B) top 10 candidate raw materials with degree. MTNR1B = melatonin receptor 1B.

Through screening, 16 compounds related to the target MTNR1B, and 86 kinds of related candidate raw materials were obtained. The network diagram constructed by the target, candidate compounds and candidate raw materials was shown in Figure 3. According to the size of the degree value, take the top 10 compounds and candidate raw materials to make a bar chart, as shown in Figure 4A and B. The annotations of the compounds are shown in Table 3. The top 5% candidate raw materials with the degree value were taken as the core raw materials. The core raw materials related to the target MTNR1B in terms of degree value were root of membranous milkvetch, reed rhizome, rhizome of gaint knotweed, sanchi, medicil evodia fruit. The results of core material screening were the same as those of target MTNR1A.

**Table 3:** Notes on compounds associated with the target MTNR1B.

Number	Compound ID	Compound name
1	HBIN014272	Absinthin
2	HBIN014467	Acetylcholine
3	HBIN014685	Adeninenucleoside
4	HBIN015253	Allyl isothiocyanate
5	HBIN021262	Colchine
6	HBIN021605	Coumari
7	HBIN021608	Coumarin
8	HBIN021843	Cucurbitacin
9	HBIN024964	Elaterin
10	HBIN026267	Evodin
11	HBIN030814	Isohumulone A
12	HBIN036930	Nigakilactone D
13	HBIN037286	(−)-Noradrenaline
14	HBIN037638	Obaculactone
15	HBIN037644	Obakulactone
16	HBIN043778	Serotonine

##### 3.1.4.3. Network construction of target HTR1A

**Figure 5.**
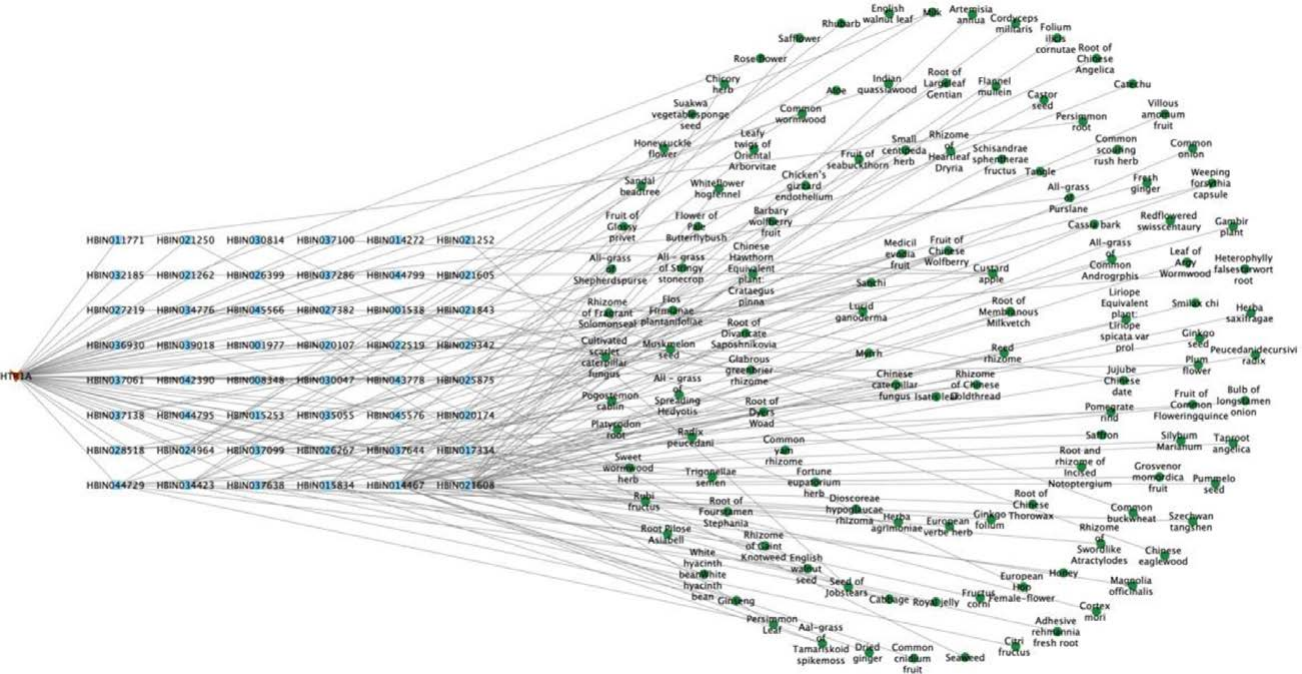
Target-compound- candidate raw materials network of HTR1A. HTR1A = 5-hydroxytryptamine receptor 1A.

**Figure 6.**
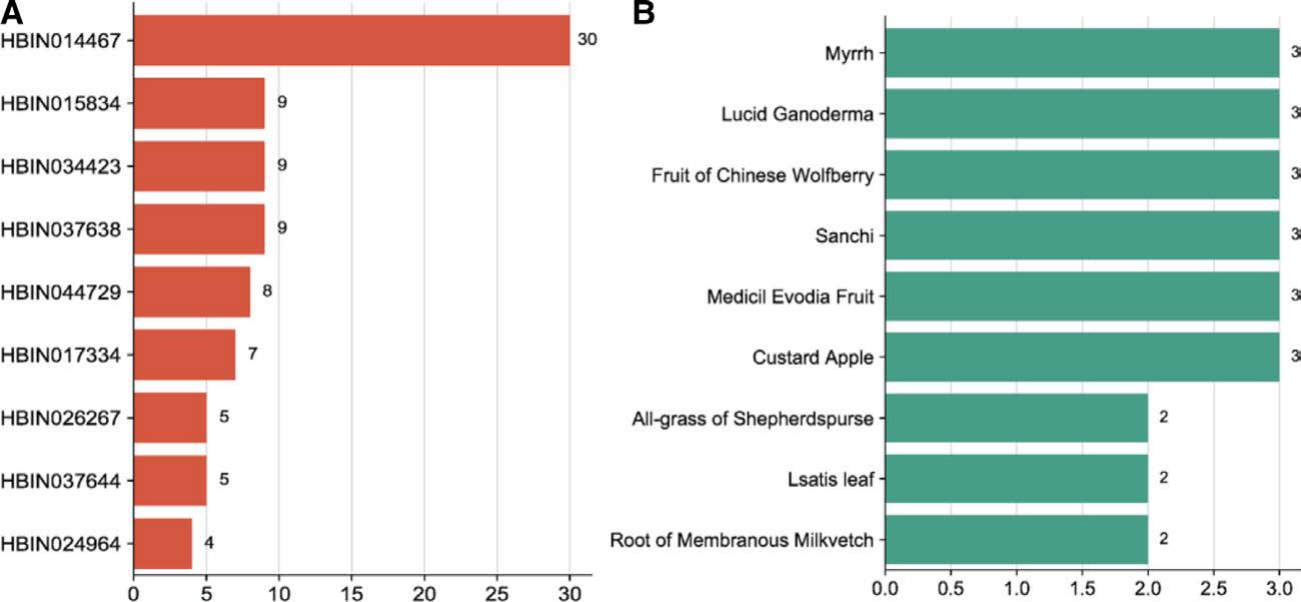
Compounds and candidate raw materials related to target HTR1A. (A) Top 10 compounds with degree; (B) top 10 candidate raw materials with degree. HTR1A = 5-hydroxytryptamine receptor 1A.

Through screening, 48 compounds related to the target HTR1A, and 120 kinds of related candidate raw materials were obtained. The network diagram constructed by the target, candidate compounds and candidate raw materials was shown in Figure 5. According to the size of the degree value, take the top 10 compounds and candidate raw materials to make a bar chart, as shown in Figure 6A and B. The top 5% candidate raw materials with the degree value were taken as the core raw materials. The core raw materials related to the target HTR1A in terms of degree value were Chinese caterpillar fungus, myrrh, lucid Ganoderma, fruit of chinese wolfberry, sanchi, medicil evodia fruit. The annotations of the compounds are shown in Table 4.

**Table 4:** Notes on compounds associated with the target HTR1A.

Number	Compound ID	Compound Name
1	HBIN001538	14-O-cinnamoylneoline
2	HBIN001977	1,7,8,10,11,12,13,14,15,17,-Decahydro-decahydro-17-(2-hydroxy-6-methylheptan-2-yl)-10,13-dimethyl-2H-cyclopenta [α] penanthrene-3(6H,9H,14H)-one
3	HBIN008348	3-Buten-2-one,4-(2,6,6-trimethyl-1-cyclohexen-1-yl)
4	HBIN011771	5-Methoxy-n-methyltryptamine
5	HBIN014272	Absinthin
6	HBIN014467	Acetylcholine
7	HBIN015253	Allyl isothiocyanate
8	HBIN015834	Amentoflavone
9	HBIN017334	Atropine
10	HBIN020107	Cephalin
11	HBIN020174	Cetylic acid
12	HBIN021250	Coixan A
13	HBIN021252	Coixan C
14	HBIN021262	Colchine
15	HBIN021605	Coumari
16	HBIN021608	Coumarin
17	HBIN021843	Cucurbitacin
18	HBIN022519	Cytidine
19	HBIN024964	Elaterin
20	HBIN025875	Ethyl aldehyde
21	HBIN026267	Evodin
22	HBIN026399	Fat
23	HBIN027219	Ganoderenic acid A
24	HBIN027382	Gastrin
25	HBIN028518	Guanine (1,7-dihydro-form)
26	HBIN029342	Hexose
27	HBIN030047	Immune globulin from
28	HBIN030814	Isohumulone A
29	HBIN032185	Kikemanine
30	HBIN034423	Mannose-B
31	HBIN034776	Mescaline
32	HBIN035055	Methyl-7-epiganoderate
33	HBIN036930	Nigakilactone d
34	HBIN037061	N-methyl-2-β-Hydroxypropyl piperidine
35	HBIN037099	(+)-N-methyl laurotetanine
36	HBIN037100	N-methyllaurotetanine
37	HBIN037138	N,N-dimethyl-5-methoxy tryptamine
38	HBIN037286	(−)-Noradrenaline
39	HBIN037638	Obaculactone
40	HBIN037644	Obakulactone
41	HBIN039018	Peanut acid
42	HBIN042390	(−)-Roemerine
43	HBIN043778	Serotonine
44	HBIN044729	Stearate
45	HBIN044795	Stephanthrine
46	HBIN044799	Stepholidine
47	HBIN045566	Tatarine
48	HBIN045576	TAU

##### 3.1.4.4. Network construction of target GABRB2

**Figure 7.**
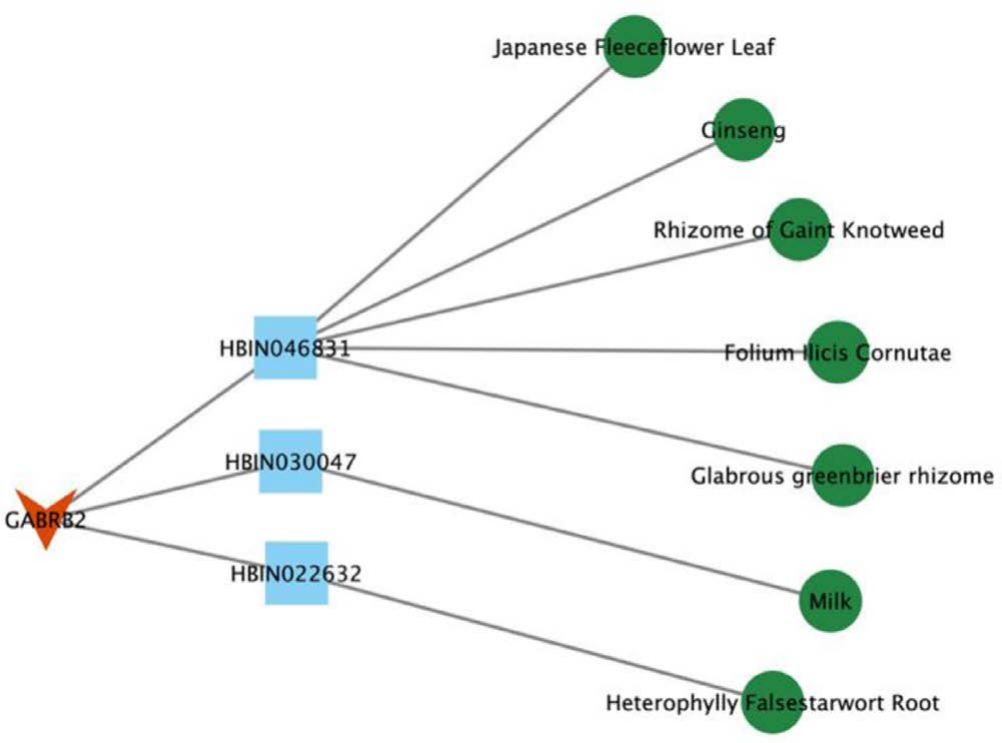
Target-compound- candidate raw materials network of target GABRB2. GABRB2 = gamma-aminobutyric acid receptor subunit beta-2.

After screening, 3 compounds related to the target GABRB2, and 7 kinds of related candidate raw materials were obtained. The network diagram constructed by the target, candidate compounds and candidate raw materials was shown in Figure 7. The annotations of the compounds are shown in Table 5. The top 5% of candidate raw materials were taken as the core raw materials, and it could be seen that rhizome of gaint knotweed was the potential core candidate raw material of target GABRB2.

**Table 5:** Notes on compounds associated with the target GABRB2.

Number	Compound ID	Compound name
1	HBIN046831	Trans-resveratrol
2	HBIN030047	Immune globulin from
3	HBIN022632	Dandelion game ethyl alcohol

##### 3.1.4.5. Network construction of target GABRA1

1264 compounds related to GABRA1, and 582 kinds of candidate raw materials were obtained by screening, and the network diagram of targets, candidate compounds and candidate raw materials was constructed. The candidate raw materials with the top 5%-degree value were taken as the core raw materials. Fresh ginger, chrysanthemum flower, myrrh, root of chinese thorowax, villous amomum fruit, perilla frutescens, coriandri sativi herba, chuanxiong rhizome, peppermint, officinal magnolia equivalent plant: magnolia bilo, leaf of argy wormwood, all-grass of haichow elsholtzia, root and rhizome of incised notoptergium, all-grass of fineleaf schizonepeta, common aucklandia root, root of ligulilobe sage, root of divaricate saposhnikovia, shrub chastetree fruit, medicil evodia fruit, honeysuckle flower, sweet wormwood herb, dried ginger, oily wood of agalloch eaglewood, taproot angelica, cassia bark, leafy twigs of oriental arborvitae, loquat leaf, fructus corni, combined spicebush root, ginkgo folium were the core raw materials. The other top 50 ingredients were shown in Table 6.

**Table 6:** Potential core candidate raw materials of target GABRA1.

Number	Materials	Degree value
1	Fresh ginger	128
2	Chrysanthemum flower	118
3	Myrrh	110
4	Root of Chinese Thorowax	102
5	Villous amomum fruit	99
6	Perilla frutescens	98
7	Coriandri sativi herba	91
8	Chuanxiong rhizome	90
9	Peppermint	86
10	Officinal Magnolia Equivalent plant: Magnolia bilo	82
11	Leaf of Argy Wormwood	77
12	All-grass of Haichow Elsholtzia	75
13	Root and rhizome of Incised Notoptergium	73
14	All-grass of Fineleaf Schizonepeta	70
15	Common aucklandia root	70
16	Root of Ligulilobe sage	64
17	Root of Divaricate Saposhnikovia	64
18	Shrub chastetree fruit	64
19	Medicil evodia fruit	60
20	Honeysuckle flower	60
21	Sweet wormwood herb	60
22	Dried ginger	59
23	Oily wood of Agalloch Eaglewood	58
24	Taproot angelica	56
25	Cassia bark	56
26	Leafy twigs of Oriental Arborvitae	56
27	Loquat leaf	55
28	Fructus corni	55
29	Combined spicebush root	54
30	Ginkgo folium	54
31	Ligustici rhizoma et radix	54
32	Fruit of Glossy privet	53
33	Rhizome of Nutgrass Galingale	53
34	Common cnidium fruit	53
35	Mulberry leaf	51
36	Pepper fruit	50
37	Rubi fructus	49
38	Pricklyash peel	49
39	Fruit of seabuckthorn	49
40	Ginseng	49
41	Clove	48
42	Alpiniae officirum rhizome	47
43	Grassleaf sweetflag rhizome	47
44	Hempleaf negundo chastetree leaf	46
45	Pogostemon cablin	44
46	Frankincense	44
47	Fortune eupatorium herb	43
48	Solidaginis herba	43
49	Zedoray rhizome	42
50	Fruit of Siberian Cockleblur	42

### 3.2. Network pharmacological analysis of the improvement of insomnia by PSAP

#### 3.2.1. Target acquisition

**Figure 8.**
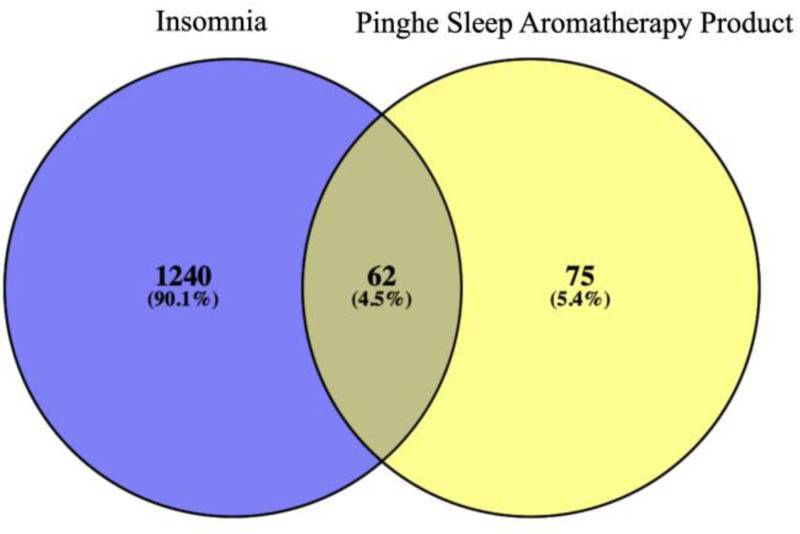
Intersection targets of insomnia and PSAP. PSAP = Pinghe Sleep Aromatherapy Product.

1302 targets of insomnia were obtained through TCMSP, Disgenet and Genecard. 137 targets of PSAP were obtained through TCMSP, Uniprot and PubMed. After taking the intersection, 62 intersection targets were obtained. As shown in Figure 8.

#### 3.2.2. String analysis

**Figure 9.**
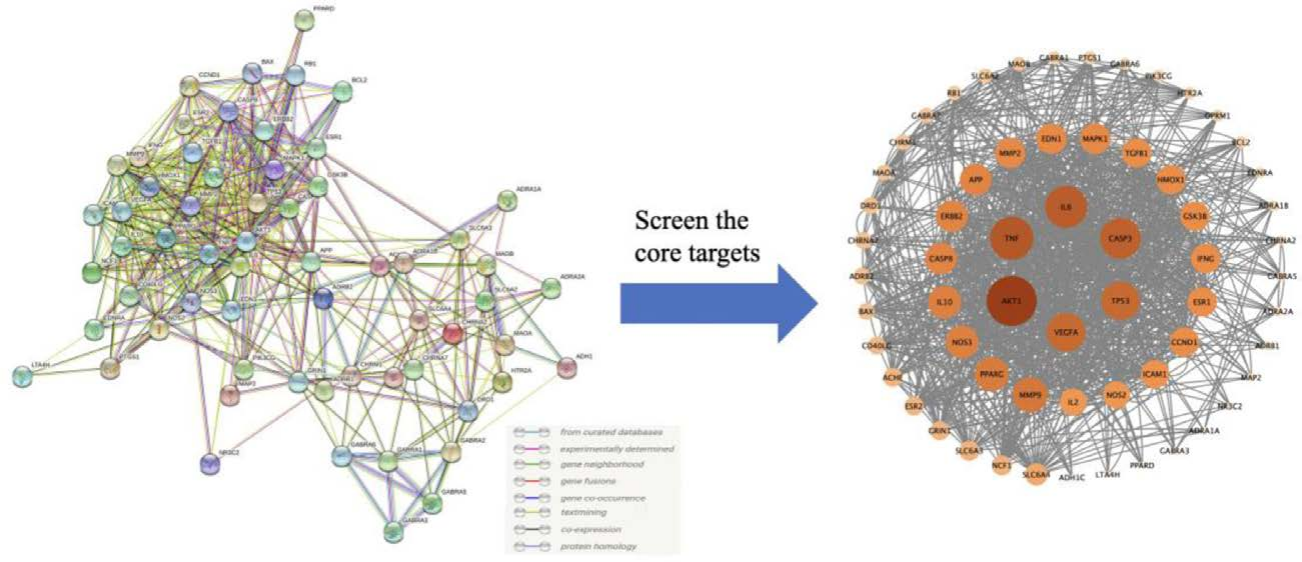
Potential core target analysis.

A total of 62 interse ction targets are imported into the Search Tool for Recurring Instances of Neighbouring Genes platform for core target analysis. A total of 25 core targets are obtained as shown in Figure 9 and Table 7.

**Table 7:** Degree value of core targets of PSAP in the treatment of insomnia.

Number	Gene	Degree value
1	AKT1	82
2	TNF	70
3	IL6	68
4	CASP3	66
5	TP53	62
6	VEGFA	62
7	MMP9	56
8	PPARG	54
9	NOS3	52
10	IL10	52
11	APP	50
12	ERBB2	50
13	CASP8	50
14	EDN1	48
15	MAPK1	48
16	MMP2	48
17	ESR1	46
18	GSK3B	46
19	CCND1	46
20	HMOX1	46
21	IFNG	46
22	TGFB1	46
23	ICAM1	44
24	NOS2	42
25	IL2	42

AKT1 = AKT serine/threonine kinase 1, APP = amyloid beta precursor protein, CASP3 = Caspase-3, CASP8 = Caspase-8, CCND1 = cyclin D1, EDN1 = endothelin 1, ERBB2 = erb-b2 receptor tyrosine kinase 2, ESR1 = estrogen receptor 1, GSK3B = glycogen synthase kinase 3 beta, HMOX1 = heme oxygenase 1, ICAM1 = intercellular adhesion molecule 1, IFNG = interferon gamma, IL10 = Interleukin 10, IL2 = interleukin 2, IL6 = Interleukin 6, MAPK1 = mitogen-activated protein kinase 1, MMP2 = matrix metallopeptidase 2, MMP9 = matrix metallopeptidase 9, NOS2 = nitric oxide synthase 2, NOS3 = nitric oxide synthase 3, PPARG = peroxisome proliferator activated receptor gamma, TGFB1 = transforming growth factor beta 1, TNF = tumor necrosis factor, TP53 = tumor protein p53, VEGFA = vascular endothelial growth factor A.

#### 3.2.3. GO and KEGG analysis

**Figure 10.**
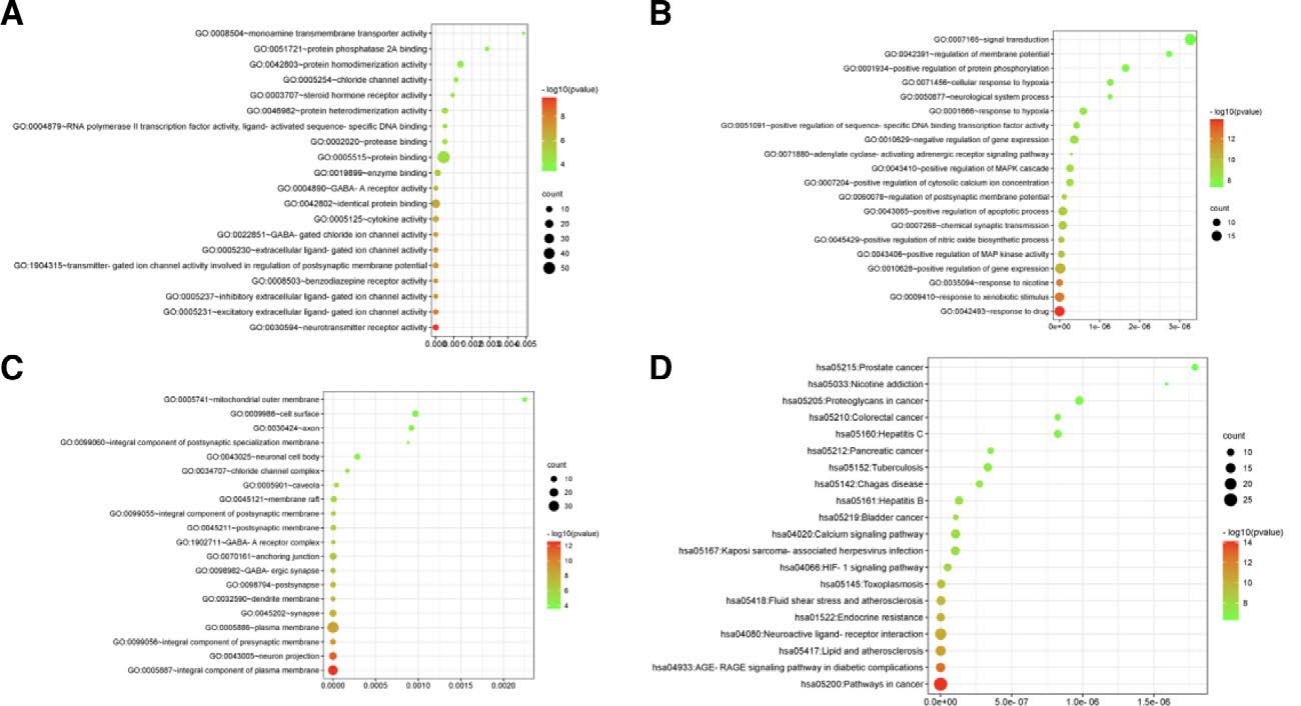
GO and KEGG analysis of PSAP in the treatment of insomnia. (A) GO-MF; (B) GO-BP; (C) GO-CC; (D) KEGG. GO = gene ontology, KEGG = Kyoto Encyclopedia of Genes and Genomes, PSAP = Pinghe Sleep Aromatherapy Product, MF = Molecular Function, BP = Biological Process, CC = Cellular Component.

GO enrichment was conducted on the core targets of the PSAP for improving insomnia. The top 20 items were selected for analysis based on the enrichment intensity (Figure 10A–C). The molecular functions are enriched in neurotransmitter receptor activity, excitatory extracellular ligand-gated ion channels, neurotransmitter gated ion channel activity involved in regulating postsynaptic membrane potential, inhibitory extracellular ligand-gated ion channels, etc. In biological processes, it is enriched in responses to drugs, responses to xenogenic stimuli, positive regulation of gene expression, and positive regulation of mitogen-activated protein kinase activity, etc. The whole of the plasma membrane, neurons, the whole of the presynaptic membrane, the plasma membrane, synapses, etc are enriched in the cell composition.

KEGG enrichment analysis was conducted on the core targets of the PSAP for improving insomnia. The top 20 pathways were selected for analysis based on enrichment intensity (Fig. 10D). The KEGG pathways closely related to insomnia include the cancer pathway, the advanced glycation end product-receptor for advanced glycation end products (AGE-RAGE) signaling pathway in diabetic complications, lipids and atherosclerosis, neuroactive ligand-receptor interactions, the hypoxia inducible factor-1 (HIF-1) signaling pathway, and the calcium signaling pathway, etc.

#### 3.2.4. Network construction

**Figure 11.**
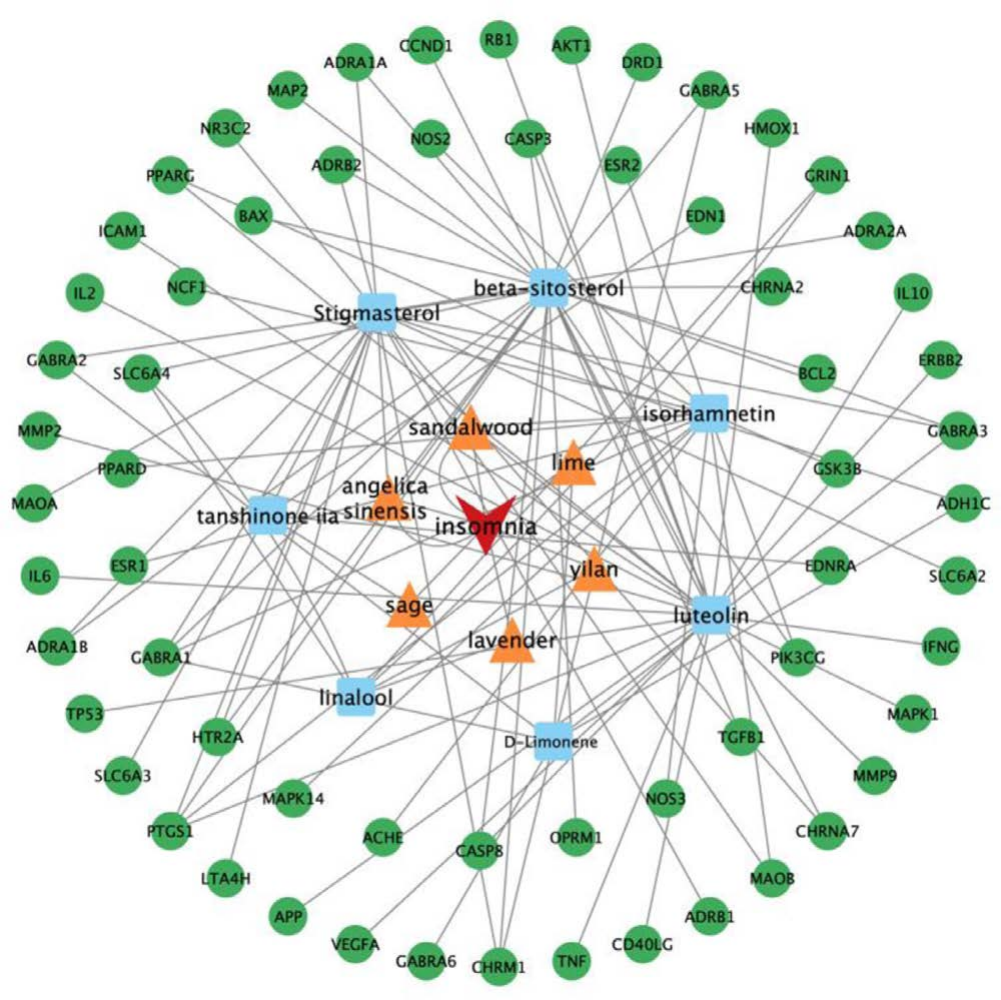
Diagram of the PSAP-key components-insomnia key targets relationship network. PSAP = Pinghe Sleep Aromatherapy Product.

Construct the PSAP-key components-insomnia key targets relationship network diagram, as shown in Figure 11. The connections between nodes represent the links among key raw materials, key components, and core targets. As can be seen from the figure, the PSAP improved insomnia through multiple pathways and targets.

## 4. Discussion

Through network analysis, the potential core candidate raw materials related to MTNR1A and MTNR1B were root of membranous milkvetch, reed rhizome, rhizome of gaint knotweed, sanchi, medicil evodia fruit. Potential core candidate raw materials related to target HTR1A were chinese caterpillar fungus, myrrh, lucid Ganoderma, fruit of chinese wolfberry, sanchi, medicil evodia fruit. A potential core candidate raw material related to GABRB2 was rhizome of gaint knotweed. Potential core candidate raw materials related to the target GABRA1 were fresh ginger, chrysanthemum flower, myrrh, root of chinese thorowax, villous amomum fruit, perilla frutescens, coriandri sativi herba, chuanxiong rhizome, peppermint, officinal magnolia equivalent plant: magnolia bilo, leaf of argy wormwood, all-grass of haichow elsholtzia, root and rhizome of incised notoptergium, all-grass of fineleaf schizonepeta, common aucklandia root, root of ligulilobe sage, root of divaricate saposhnikovia, shrub chastetree fruit, medicil evodia fruit, honeysuckle flower, sweet wormwood herb, dried ginger, oily wood of agalloch eaglewood, taproot angelica, cassia bark, leafy twigs of oriental arborvitae, loquat leaf, fructus corni, combined spicebush root, ginkgo folium.

The potential core candidate raw materials of target MTNR1A and target MTNR1B are the same. The potential core candidate raw materials shared by target MTNR1A, target MTNR1B and target HTR1A were sanchi, medicil evodia fruit. The potential core candidate raw material shared by target MTNR1A, target MTNR1B and target GABRB2 was rhizome of gaint knotweed. The potential core candidate raw material shared by target MTNR1A, target MTNR1B, target HTR1A and target GABRA1 was medicil evodia fruit.

Melatonin is an important endocrine hormone, mainly secreted by the pineal gland and the retina, and acts by binding to the melatonin receptor, which has the function of regulating physiological rhythm, sleep, and reproduction. Studies have shown that the MTNR1A and the MTNR1B is expressed on the surface of nervous system, pancreatic β cells, liver, skeletal muscle, adipose tissue, and ovarian granulosa cells.^[12,13]^ The variation of melatonin receptor is the cause of human sleep disorders, polycystic ovary syndrome, gestational diabetes, recurrent kidney stones and other diseases.^[14]^ Ebisawa et al^[15]^ found that there were 7 mutations in the MTNR1A gene between patients with circadian dysrhythmic sleep disorder and healthy people. Park et al^[16]^ found that SNP rs2119882 in the human MTNR1A promoter region is associated with schizophrenia and its resulting insomnia. MTNR1B receptor is involved in the pathophysiological and pharmacological processes of sleep disorders, anxiety, depression, Alzheimer disease, and pain.^[17]^

Serotonin, also known as 5-hydroxytryptamine, plays an important role in the regulation of animal behavior. HTR1A is an inhibitory G-protein-coupled receptor that is expressed on both serotonin and non-serotonin neurons in mammals.^[18]^ Studies have shown that HTR1A plays an important role in mental disorders^[19]^ and is the main target of most antidepressant drugs.^[20]^ When activated by serotonin, HTR1A can regulate mood and relieve anxiety.^[18]^ Wang et al found that Chaihu-Longgu-Muli Decoction can enhance the expression of HTR1A, regulate the secretion of neurotransmitters, and have sedative and hypnotic effects.^[21]^ Sun et al^[22]^ found that Banxia-Houpo decoction remedy ameliorates corticosterone-induced depressive behaviors in mice via targeting 5-hydroxytryptamine receptor 1A signaling.

Gamma-aminobutyric acid A receptor (GABAAR) is a ligand-gated chloride ion channel protein that mediates the main inhibitory function of the central nervous system, and its dysfunction plays an important role in the etiology of epilepsy.^[23,24]^ GABRA1, a member of the GABAAR subunit family, encodes the α1 subunit of GABAAR, and its coding gene is located on human chromosomes 5q34-q35, which is related to mood disorders.^[25]^ The GABRB1 gene encodes the β1 subunit of GABAAR, which is responsible for mediating inhibitory neurotransmission in the thalamus.^[26]^ Studies have found that downregulation of the GABA receptor subunits is positively correlated with schizophrenia.^[27]^ Upregulating the expression of γ-aminobutyric acid receptors can increase the opening frequency of chloride ion channels and play a sedative and hypnotic role.^[28]^

Root of membranous milkvetch is a representative drug for supplementing qi and strengthening surface, and called “the length of tonic medicine” in Compendium of Materia Medica.^[29]^ Modern studies have shown that root of membranous milkvetch has many biological activities, such as wound healing, immune regulation, antitumor, liver protection, antimutagenesis and so on.^[30]^ As the most widely used qi tonic drug in clinic, root of membranous milkvetch can be used to modulate immune function^[31]^ and is a common drug for improving insomnia.^[32]^ Wang et al^[33]^ found that astragalus could improve the cerebral artery blood flow speed, alleviate cerebral tissue ischemia, and prolong sleep time.

Reed rhizome is a grass plant of the genus Phragmites communis Trin. It is a variety collected in the “Chinese Pharmacopoeia.” It is a commonly used traditional Chinese medicine with the same origin of medicine and food. It is cold in nature and sweet in taste and has the effects of clearing heat and promoting fluid, eliminating irritability, stopping vomiting and diuresis.^[34]^ Modern pharmacological studies have shown that reed rhizome has antioxidant, hypoglycemic, antitumor, liver protection and other effects.^[35]^ The lignan glycosides, flavonoids and phenylpropanoids contained in reed rhizome have antioxidant activity and α-glucosidase inhibitory activity.^[36]^

Rhizome of gaint knotweed is the dried rhizome and root of *Polygonum cuspidatum* sieb.et Zucc. containing polydatin, resveratrol, emodin, etc.^[37]^ Rhizome of gaint knotweed has pharmacological effects such as anti-inflammation, antioxidation, regulating blood lipids and improving cognition.^[38,39]^ Rhizome of gaint knotweed can significantly improve the neurological dysfunction of mice with traumatic brain injury, alleviate brain edema and reduce neuronal degeneration, enhance neuronal autophagy and inhibit neuronal apoptosis.^[40]^

Sanchi is a dried root and rhizome of *Panax notoginseng* (burkill) F.H. Chenexc.Chow&W.G.Huang, which has the effects of dispelling stasis, stopping bleeding, reducing swelling and relieving pain.^[41]^ Sanchi mainly contains a variety of pharmacodynamic components such as total notoginseng saponins, notoginseng pigments and flavonoids,^[42]^ which can promote blood circulation, stop bleeding, antithrombus and enhance body immunity.^[43]^

Medicil evodia fruit is a genus of rutaceae, mainly distributed in the south of Qinling Mountains in China, especially in Yungui and Chuanshu regions, and uses dried and nearly ripe fruits as medicine.^[44]^ The taste is pungent, bitter, hot, and small poison.^[34]^ Medicil evodia fruit mainly contains alkaloids, picrogens, flavonoids and other chemical components.^[45]^ Studies have shown that medicil evodia fruit can reduce the levels of inflammatory factors such as tumor necrosis factor alpha (TNF-α) and interleukin 1 beta (IL-1β),^[46]^ increase the activity of superoxide dismutase (SOD) and glutathione peroxidase (GSH-Px) in brain, and reduce the production of free radicals, reduce the content of nitric oxide (NO) and the activities of nitric oxide synthase (NOS) and inducible nitric oxide synthase (iNOS).^[47]^

The plant raw materials mentioned above may have the potential to improve insomnia. Plant raw materials with multiple targets of action may be important because of their potential broad-spectrum effects on insomnia. Combining multiple plant raw materials associated with multiple targets may be an improvement strategy.

Based on the screening results, we selected sandalwood, lime, angelica sinensis, yilan, sage and lavender to design PSAP to improve insomnia. Analysis reveals that the medicinal ingredients in the PSAP such as beta-sitosterol, stigmasterol, isorhamnetin, luteolin, tanshinone IIA, D-limonene, and linalool can act on target genes such as IL6, TNF, AKT1, CASP3, TP53, and VEGFA, regulate signaling pathways such as AGE-RAGE, neuroactive ligand-receptor interactions, the HIF-1 signaling pathway, and the calcium signaling pathway, etc, thereby exerting the effect of improving insomnia. Its mechanism of action involves multiple pharmacological components, target genes and signaling pathways.

## 5. Conclusion

In this study, a target-compound raw material network was constructed based on network pharmacology and data mining. Core natural raw materials that may help to improve insomnia were identified by network, providing a theoretical basis for the development of aromatic products to improve insomnia. Based on the screening results, we developed the PSAP. However, the potential target genes and signaling pathways of the PSAP explored in this study for improving insomnia still need to be further verified by relevant experiments. But in general, this study provides the data foundation for product development, and provides an idea of raw material screening and product development. Using network pharmacology and data mining, product development costs can be saved, and product development cycle can be shortened.

## Author contributions

**Conceptualization:** Yan Jia.

**Data curation:** Qian Jiao.

**Formal analysis:** Meiting Yi.

**Project administration:** Congfen He.

**Writing – original draft:** Lizhi Yue.

**Writing – review & editing:** Junxiang Li, Yi Lu.
